# The effect of early versus late treatment initiation after diagnosis on the outcomes of patients treated for multidrug-resistant tuberculosis: a systematic review

**DOI:** 10.1186/s12879-016-1524-0

**Published:** 2016-05-04

**Authors:** Rebecca C. Harris, Louis Grandjean, Laura J. Martin, Alexander J. P. Miller, Joseph-Egre N. Nkang, Victoria Allen, Mishal S. Khan, Katherine Fielding, David A. J. Moore

**Affiliations:** TB Centre, London School of Hygiene and Tropical Medicine, Keppel Street, London, WC1E 7HT UK; Department of Infection, Immunology and Rheumatology, University College London, Institute of Child Health, Guilford Street, London, WC1E 6BT UK; Royal Brompton and Harefield NHS Foundation Trust, Sydney Street, London, SW3 6NP UK; Chelsea and Westminster Hospital, 369 Fulham Road, London, SW10 9NH UK; Saw Swee Hock School of Public Health, National University of Singapore, Singapore, 119077 Singapore; The School of Public Health, University of the Witwatersrand, Johannesburg, South Africa

**Keywords:** Multi-drug resistant, Extensively drug resistant, Tuberculosis, Treatment delay, Systematic review

## Abstract

**Background:**

Globally it is estimated that 480 000 people developed multidrug-resistant tuberculosis (MDR-TB) in 2014 and 190 000 people died from the disease. Successful treatment outcomes are achieved in only 50 % of patients with MDR-TB, compared to 86 % for drug susceptible disease. It is widely held that delay in time to initiation of treatment for MDR-TB is an important predictor of treatment outcome. The objective of this review was to assess the existing evidence on the outcomes of multidrug- and extensively drug-resistant tuberculosis patients treated early (≤4 weeks) versus late (>4 weeks) after diagnosis of drug resistance.

**Methods:**

Eight sources providing access to 17 globally representative electronic health care databases, indexes, sources of evidence-based reviews and grey literature were searched using terms incorporating time to treatment and MDR-TB. Two-stage sifting in duplicate was employed to assess studies against pre-specified inclusion and exclusion criteria. Only those articles reporting WHO-defined treatment outcomes were considered for inclusion. Articles reporting on fewer than 10 patients, published before 1990, or without a comparison of outcomes in patient groups experiencing different delays to treatment initiation were excluded.

**Results:**

The initial search yielded 1978 references, of which 1475 unique references remained after removal of duplicates and 28 articles published pre-1990. After title and abstract sifting, 64 papers underwent full text review. None of these articles fulfilled the criteria for inclusion in the review.

**Conclusions:**

Whilst there is an inherent logic in the theory that treatment delay will lead to poorer treatment outcomes, no published evidence was identified in this systematic review to support this hypothesis. Reports of programmatic changes leading to reductions in treatment delay exist in the literature, but attribution of differences in outcomes specifically to treatment delay is confounded by other contemporaneous changes. Further primary research on this question is not considered a high priority use of limited resources, though where data are available, improved reporting of outcomes by time to treatment should be encouraged.

**Electronic supplementary material:**

The online version of this article (doi:10.1186/s12879-016-1524-0) contains supplementary material, which is available to authorized users.

## Background

The widespread emergence of multidrug-resistant tuberculosis (MDR-TB) and extensively drug-resistant tuberculosis (XDR-TB) could limit the globally declining trend in tuberculosis (TB) prevalence that has been observed in recent years. It is estimated that worldwide 20 % of previously treated TB cases and 3.3 % of new TB cases now have multidrug-resistant tuberculosis – caused by bacterial strains resistant to at least isoniazid and rifampicin [1].

Treatment for MDR-TB and XDR-TB currently entails therapeutic regimens with much lower efficacy and greater toxicity than those used for drug-susceptible TB. Successful treatment outcome was only reported for 50 % of MDR-TB patients globally in 2014, compared to 86 % for newly diagnosed drug susceptible disease [1], and second line agents commonly used are poorly tolerated. Current recommendations for treatment of MDR-TB require at least 20 months of therapy [2], though mounting evidence indicates that shorter regimens may perform at least as well [3].

Early treatment of MDR-TB is presumed to be associated with improved treatment outcomes, yet the evidence in support of this assumption has not been previously reviewed. Individuals with a prolonged delay to treatment are perceived as more likely to have a higher bacillary burden, more extensive lung damage and, as a result, active TB disease that is harder to treat. With the introduction of rapid diagnostic tools and reported reductions in time to identification and treatment of MDR-TB in many settings, there is interest in determining whether such reductions are associated downstream with improved MDR-TB treatment outcomes. Moreover some countries are struggling to keep up with the demand for MDR treatment as they diagnose more and more cases, creating “waiting lists” wherein patients have to wait until treatment capacity is available. Demonstration of an adverse effect upon patient outcome of such delays would be a potentially useful tool for strengthened advocacy to promote interventions that enhance linkage of test results to treatment.

We therefore undertook a systematic review of the existing evidence on the outcomes of multidrug-resistant and extensively drug-resistant tuberculosis patients treated early (≤4 weeks) versus late (>4 weeks) after the diagnosis of drug resistance. This review contributes to the evidence base for the generation of an updated World Health Organization (WHO) guideline for the clinical management of MDR-TB and XDR-TB.

## Methods

### PICOT Question

The full original protocol and PRISMA checklist [4] (Additional file 1: Table S1) are available in the supporting information additional file section. The following amendments or clarifications were made to the original protocol: searching Google Scholar in place of Google, including a limit of publication since 1990, including a 10 % check of sifting by a third reviewer, and allowing a flexible cut-off during sifting in the definition of the timing of early versus late treatment.

The research question was framed using PICOT (Population, Intervention, Comparison, Outcome and Time) methodology [5, 6]. The population under consideration was all patients with multidrug-resistant or extensively drug-resistant tuberculosis bacteriologically confirmed by phenotypic methods, or for whom molecular testing indicating rifampicin resistance was used as a surrogate for initiation of MDR treatment. The intervention and comparator of interest were early versus late treatment, defined respectively as drug treatment initiated within 28 days versus later than 28 days after diagnosis of MDR-TB. The primary outcomes in this review were the WHO-defined tuberculosis outcomes as proposed by Laserson et al. [7]: cure, treatment completion, failure/relapse, transfer out, abandoned treatment and death. Treatment success was defined as patients meeting the outcome definition of cure or treatment completed. Poor outcomes were defined as patients with the outcome of failure/relapse, transfer out, abandoned treatment or death. Secondary outcomes pre-defined in the protocol were not included in the search terms, but were intended for consideration during data extraction from included papers, and were defined as adverse reactions from TB drugs (severity, type, organ class), adherence to treatment, or treatment interruption due to non-adherence.

### Search strategy

A comprehensive search strategy was developed in consultation with WHO technical experts using the PICOT question as a framework (see Additional file 1). Due to the relatively low number of total hits in preliminary searches, only population and intervention terms were used in the search strategy (Additional file 1: Table S2). By checking for comparator and outcomes defined a priori during the manual sift instead of in the search strategy, the likelihood of missing a potentially relevant paper was reduced. An example of the search strategy, in this case as applied in PubMed, is included in Additional file 1: Table S2.

Electronic health care databases, sources of evidence-based reviews, guidelines, and grey literature were searched in accordance with the specifications of each database. These included PubMed (including MEDLINE), EMBASE, Cochrane library (includes CENTRAL, CDSR, DARE and HTA databases), WHO Global Index Medicus (includes LILACS, WPRIM, IMSEAR, IMEMR, AIM, SciELO and WHOLIS indexes), WHO portal of clinical trials, OpenSIGLE, International Union of Tuberculosis and Lung Disease conference abstracts (2004–2014) and Google Scholar (limited to the first ten pages). The search strategies were executed on 26th September 2015. The date, human studies and language inclusion criteria were applied in the manual sifting process instead of through the limits function of PubMed (and other databases) in order to avoid exclusion of papers that had not been indexed on these criteria (Table 1).

**Table 1:** Summary of non-PICO inclusion criteria

Limit category	Specified limit	Implementation
Languages	English, Japanese, Chinese, Russian, French, Spanish, Portuguese, Ukrainian, Lithuanian	Manual sifting
Publication type	None	n/a
Date of publication	1st January 1990–26th Sept 2015	Manual sifting
Study design	Consecutive Case Series, Case Control Studies, Cohort Studies, Randomised Controlled Trials, Systematic Reviews and Meta-analyses	Manual sifting
Other limits	None	n/a

### Manual sifting

Following removal of duplicates, two-stage screening against inclusion and exclusion criteria was executed independently by two reviewers (JN, AM), sifting first by title and abstract, followed by full-text sifting. A third reviewer (DM) reviewed any discrepant results and every tenth reference to adjudicate and check for consistency. Studies including participants of any age with confirmed multi-drug resistant or extensively drug-resistant pulmonary tuberculosis were potentially eligible for inclusion. Any consecutive case series, case control study, cohort study, randomised controlled trial, systematic review or meta-analysis that included a comparator group was considered for inclusion. Included studies were required to report data on at least one of the primary outcome measures of interest. Although the original intervention of interest was defined as presumed adequate treatment initiated ≤28 days after diagnosis, and a comparator group with treatment initiated >28 days after diagnosis, the protocol was changed to allow flexibility in the definition of this cut off for early versus late treatment. Therefore studies reporting outcomes for patient groups with different definitions for delay to treatment initiation were considered for inclusion.

Any systematic review superseded by an updated systematic review, or narrative reviews not adding new data or new analysis to the existing evidence base were excluded. Finally, studies not performed in humans, written in a language other than those listed in Table 1, or with fewer than ten participants were excluded.

Sifting was primarily managed within Endnote® X7.4 (Thomson Reuters, California, USA). If a paper was deemed ineligible for inclusion at the full text sifting stage, the primary reason for ineligibility was recorded by the reviewers.

### Supplementary review and data extraction

An additional post-hoc review of those articles included in the full text review was undertaken by one reviewer (DM) using alternative, less-stringent inclusion criteria. Specifically, papers were identified which reported on two or more groups of patients with different times to MDR treatment initiation. Data on treatment outcome according to WHO criteria or on intermediate outcomes such as culture conversion time (though not validated as surrogates of treatment outcome) were abstracted from papers included in this post-hoc review.

## Results

**Fig. 1:**
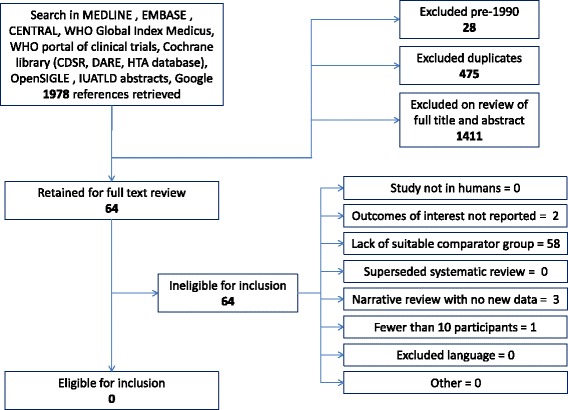
Flow chart summarising search results

A total of 1978 citations were retrieved from the initial search of all databases. Breakdown by database source is provided in Additional file 1: Table S3. After removal of duplicates (*n* = 475) and pre-1990 publications (*n* = 28), 1475 unique citations remained. 1411 hits were excluded during title and abstract screening. Sixty-four references were thus identified for full text review and all were retrieved [3, 8–70] (Fig. 1).

On full text review none of the 64 references fulfilled the per-protocol inclusion criteria. Reasons for exclusion are indicated in Fig. 1.

The most frequent reason for exclusion was the lack of a comparator group (91 % of full texts reviewed). Papers often reported average time to treatment, but did not disaggregate outcomes by timing of treatment, even with the more relaxed early versus late definition (not requiring a 28 day cut-off). Three narrative reviews were excluded as no new data were presented in addition to not meeting other inclusion/exclusion criteria for this review, two research articles were excluded as there were no reported outcomes of interest, and one reported on fewer than ten participants.

### Supplementary post-hoc review

Although the full text review failed to deliver any publications fulfilling the inclusion criteria it was noted that a number of articles reported less well defined data on treatment delay (without a 28 day cut-off) related to some interim outcome measures (smear and culture conversion) and final treatment outcomes [11, 13, 22, 31, 37, 38, 45, 46, 48, 50, 51, 61, 63, 64, 66, 68].

None of these articles addressed the independent effect of treatment delay with a meaningful comparator group - whether not delayed or less delayed - upon treatment outcomes, whether interim or final.

Whilst outside the designated protocol we proceeded to abstract these data in case the resulting narrative yielded any information of use. These data and narrative summary are reported in Table 2 and Additional file 1: supplementary text.

**Table 2:** Supplementary post-hoc review data

Author	Journal	Year	Exposure	Outcome	Comments
Goble [38]	NEJM	1993	Duration of disease	Failure: continually positive sputum cultures after at least three months of therapy	Duration of disease very long
			1-3 yrs	12/44	
			4-8 yrs	18/44 OR 1.8 (0.6-5.4)	
			≥9 yrs	17/46 OR 1.6 (0.5-5.0)	
Chan [37]	AJRCCM	2004	Each additional year delay before first visit to site	Initial favourable response: ≥3 negative sputum cultures over ≥3 months OR 0.93 (0.87-0.995) p=0.03	Median pre-therapy disease duration = 4.2 years; analysis takes no account of time to MDR therapy, just time to first visit
Bonilla [66]	PLoS ONE	2008		Treatment success	Paper mainly about individualisation of regimens with DST and availability of 2^nd^ line DST within 31 days; no data on lead-in time from diagnosis and exclusions from primary analyses limit interpretation
			MDR DST available within ≤31 days	264/334 (79.0%)	
			MDR DST available after > 31 days	108/160 (67.5%)	
			XDR DST available within ≤31 days	11/14 (78.6%)	
			XDR DST available after > 31 days	7/23 (30.4%)	
Dheda [31]	Lancet	2010	Treatment outcome	Delay to treatment	Compared delay to treatment in groups of survivors and non-survivors and culture converters and non-converters. Delay to treatment = time from sputum acquisition to start of treatment
			Survival	78 days [53–107]	
			Death	57 days [36–67] p=0·001	
			Culture conversion	91 days [61–116]	
			Non-conversion	59 days [43–86] p=0·001	
Heller [45]	IJTLD	2010		Median days (95%CI) treatment delay	Before vs. after comparison following change from traditional hospital based management (TM) to community based (CM). In multivariate analysis time to smear conversion was longer for TM group than for CM group (aHR=1.78, p=0.062), as was time to culture conversion (aHR=1.82, p=0.026)
			Traditional (n=46)	106.5 (88.6-151.1)	
			Community (n=48)	84 (78.7-93.3) p=0.002	
				Median days (95%CI) to smear conversion	
			Traditional (n=48)	91 (72.2-119.8)	
			Community (n=32)	59 (34.9-83.1) p=0.055	
				Median days (95%CI) to culture conversion	
			Traditional (n=53)	119 (106.1-131.9)	
			Community (n=39)	85 (68.0-102.0) p=0.002	
				Active and on treatment at 6 months	
			Traditional	91.2%	
			Community	84.8% p=0.4	
Seddon [64]	CID	2012	Treatment delay (not defined)	Not associated with: [1] failure to culture convert by month 2 (26/74, p=0.25) [2] unfavourable treatment outcome (15/103, p=0.36) [3] death (8/103, p=0.18)	Median delay 91 days (IQR 51–166) Data in table 4 – analysis not clear
Van der Walt [13]	ERJ (Conference abstract)	2012		Time to treatment	Shorter time to treatment in inpatients but no differences in time to smear or culture conversion
			Inpatients	76 days	
			Community	64 days p<0.01	
				Sputum conversion	
			Inpatients	54%	
			Community	52%	
				Time to conversion (median with IQR)	
			Inpatients	105 (64.5-164)	
			Community	121 (61.0-206.5)	
Loveday [46]	IJTLD	2012		Median (IQR) treatment delay in days	Decentralised vs. centralised hospital care. Shorter delay to treatment but worse treatment outcomes for decentralised care, but many other differences in care beyond treatment initiation delay.
			Decentralised	72 (56–99)	
			Centralised	93 (71–120) p<0.001	
				Unsuccessful treatment outcomes	
			Decentralised	96/419 (23%)	
			Centralised	37/441 (8%)	
Cox [63]	IJTLD	2014		Median (IQR) treatment delay in days	MDR programme implemented. But changes other than treatment initiation delay e.g. change to include moxifloxacin
			Before (2005)	58 (25–91) (n=39)	
			During (2010)	31 (18–45) (n=183)	
				Treatment success	
			Before (2005–7)	85/206 (41%)	
			During (2010)	86/164 (52%)	
Mpagama [48]	PLoS ONE	2013	Median (range) time from MDR diagnosis to treatment	Outcome	No difference in time from MDR diagnosis to treatment initiation between intensive phase completers and deaths.
			272 (26–888)	Completion of intensive phase n=54	
			255 (193–317)	Died n=4 p=0.8	
Chan [50]	PLoS ONE	2013	Delay	Treatment success in 3 models Multiple logistic analysis	Change to programme management in Taiwan
			>120 days	133/194 (69%)	
			≤120 days	328/457 (72%)	
			>120 vs.≤120	OR 1.2 (0.8-1.7), p=0.4 Adjusted HRs 0.8 (0.6-0.9), p=0.012 0.8 (0.6-1.0), p=0.018	
			Delay in 390 patients with second line drug susceptibility testing		
			>120 days	74/117 (63%)	
			≤120 days	170/273 (62%)	
			>120 vs. ≤120	OR 1.0 (0.6-1.5), p=0.9 aOR 0.6 (0.4-0.9), p=0.01	
Helbling [61]	Swiss Med Wkly	2014	Time to treatment	Treatment success 39/51 (76.5%) Time to treatment initiation not associated with treatment success in logistic regression model (no data shown)	Median time to initiation was 5.5 weeks but 10 initiated MDR treatment immediately
Kipiani [68]	CID	2014	Line probe assay implementation	Delay to MDR treatment	Before vs. after analysis of line probe assay implementation. Groups differed in many ways – post implementation group had more HCV co-infection, more initial inpatient treatment, more likely to receive kanamycin instead of capreomycin, higher rates on prior MDR treatment, resistant to more drugs.
			Pre-implementation	83.9 (56–106)	
			Post-implementation	18.2 (11–24) p<0.01 (Unclear if overall or just for subset who received first line drugs)	
				12 wk culture conversion	
			Pre-implementation	5/68 (7%)	
			Post-implementation	25/51 (49%)	
				24 week culture conversion	
			Pre-implementation	43/68 (63%)	
			Post-implementation	44/51 (86%) p=0.01	
				24 week smear conversion	
			Pre-implementation	77%	
			Post-implementation	90% p=0.05	
Li [51]	Lancet Global Health	2015	Programme implementation	Median [IQR] time to treatment	Time to treatment only reported for 32% and 71% of pre- and post-intervention patients
			Before	139 [69–207]	
			After	14 [10–21]	
				Still on treatment at 6 months	
			Before	8% (2/26)	
			After	80% (137/172)	
Loveday [11]	IJTLD	2015		Median (IQR) treatment delay in days	Includes all of Loveday 2012 data plus data for 7 additional months
			Decentralised	72 (54–97) (n=724)	
			Decentralised	72 (54–97) (n=724)	
			Centralised	92 (69–120) (n=811) p<0.001	
				Treatment success	
			Decentralised	427/736 (58%)	
			Centralised	439/813 (54%) p=0.18	
				Death	
			Decentralised	133/736 (18.1%)	
			Centralised	113/813 (13.9%) p=0.21	
Otero [22]	TMIH	2015	Treatment outcomes	Median (IQR) time in days to MDR-TB treatment	Should be noted that the duration of treatment prior to switching was undetermined.
			For patients starting on MDR regimen:		
			Success	26 (18–41)	
			Not success	25 (18–30) p=0.6	
			For patients switching to MDR regimen:		
			Success	11.5 (2–35)	
			Not success	22 (2–48) p=0.1	

## Discussion

Whilst there is an inherent logic in the theory that treatment delay will lead, via more severe disease, to worse treatment outcomes, we were unable to find any published evidence to support this assumption. More pertinently, there were no published data demonstrating an independent effect upon treatment outcome of earlier initiation of therapy following MDR diagnosis.

It is unfortunate that such data do not exist, as evidence highlighting a clear direct benefit upon patient-centred outcomes could have provided a powerful tool to advocate for specific interventions to improve linkage of MDR diagnosis to initiation of treatment, a particular problem for countries facing growing “waiting lists” of patients diagnosed with MDR-TB awaiting health system capacity to deliver treatment, or with considerable loss to follow up between testing and initiation of treatment. This systematic review found a lack of data rather than a lack of effect, therefore it should not be assumed that there is no benefit of early access to treatment. However, a recent systematic review evaluating the potential benefit of active case finding upon treatment outcomes for drug susceptible TB failed to demonstrate any improvement over passive case finding, despite the tendency of active case finding to find cases earlier and with reduced disease severity [71].

Regardless, beyond the effect for the individual, earlier treatment initiation should de facto result in reduced duration of infectiousness and thus result in reduced transmission at a community level. Treatment for MDR-TB should clearly be initiated at the earliest opportunity after diagnosis.

A major obstacle in this review was the lack of suitable comparator group, as no studies reported treatment outcomes for otherwise similar patient groups varying only the time to treatment. Where outcome data were reported related to time to treatment, it was often confounded by simultaneous changes in other elements of healthcare delivery, such as programmatic changes related to delivery of care and altered drug regimens. Therefore, it was impossible to attribute differential outcomes to treatment delay.

The authors believe that further research with time to treatment as the primary research question should not be considered a high priority amongst competing demands upon limited resources. However, improved data collection and reporting of patient outcomes by time to treatment initiation should be encouraged in studies collecting data on the outcomes of interest in this patient group. Such data could provide important insight without cost implications.

Even if such data were available, treatment delay when defined as the interval from MDR diagnosis to treatment fails to take account of delays in reaching an MDR diagnosis, the effect of which might overwhelm any potential benefit of reduced diagnosis-to-treatment time. Roll-out of new rapid diagnostics, whether molecular tests such as Xpert MTB/RIF or Genotype MTB DR-plus or direct phenotypic tests such as MODS or the nitrate reductase assay, is designed to reduce such delays through improved access and faster laboratory turnaround. Data comprehensively demonstrating an effect upon outcome of shortened time to MDR diagnosis are still awaited.

## Conclusion

There is currently no published evidence available to assess the effect of early versus late treatment initiation upon the outcomes of patients treated for MDR-TB or XDR-TB. Whilst supportive evidence would have provided a useful advocacy tool, we feel that the intuitive logic and inherent biological plausibility mean that MDR treatment should be initiated promptly. Initiating primary research for this research question is not considered a priority amongst competing demands upon limited resources. However, improved collection of data on time to treatment initiation and treatment outcomes within other studies could provide insight into this question.

## Ethics approval and consent to participate

Not Applicable.

## Availability of data and materials

No data were identified for inclusion in the main review. The dataset supporting the post-hoc review in this article is included within the article in Table 2 and in the Additional file 1: supplementary text.

## Additional file

Additional file 1: Table S1. PRISMA checklist. **Table S2**: Search terms applied in PubMed. **Table S3**: Number of search hits by database source. Supplementary post-hoc review text. (DOCX 139 kb)

## Abbreviations

MDR-TB: multidrug-resistant tuberculosis
PICOT: population, intervention, comparison, outcome and time
TB: tuberculosis
WHO: World Health Organization
XDR-TB: extensively drug resistant tuberculosis
