# Supplementary material for: The effect of early versus late treatment initiation after diagnosis on the outcomes of patients treated for multidrug-resistant tuberculosis: a systematic review
Source: BMC Infect Dis. 2016 May 4;16:193. doi: 10.1186/s12879-016-1524-0 (PMC4855810; doi:10.1186/s12879-016-1524-0)
Supplement: Additional file 1: Table S1. — PRISMA checklist. Table S2: Search terms applied in PubMed. Table S3: Number of search hits by database source. Supplementary post-hoc review text. (DOCX 139 kb) [file 12879_2016_1524_MOESM1_ESM.docx]

**Supplementary Information**

Table S1: PRISMA checklist

| **Section/topic** | **#** | **Checklist item** | **Reported on page #** |
| --- | --- | --- | --- |
| **TITLE** | | |  |
| Title | 1 | Identify the report as a systematic review, meta-analysis, or both. | 1 |
| **ABSTRACT** | | |  |
| Structured summary | 2 | Provide a structured summary including, as applicable: background; objectives; data sources; study eligibility criteria, participants, and interventions; study appraisal and synthesis methods; results; limitations; conclusions and implications of key findings; systematic review registration number. | 2-3 |
| **INTRODUCTION** | | |  |
| Rationale | 3 | Describe the rationale for the review in the context of what is already known. | 4 |
| Objectives | 4 | Provide an explicit statement of questions being addressed with reference to participants, interventions, comparisons, outcomes, and study design (PICOS). | 5-6 |
| **METHODS** | | |  |
| Protocol and registration | 5 | Indicate if a review protocol exists, if and where it can be accessed (e.g., Web address), and, if available, provide registration information including registration number. | 5, supporting information |
| Eligibility criteria | 6 | Specify study characteristics (e.g., PICOS, length of follow-up) and report characteristics (e.g., years considered, language, publication status) used as criteria for eligibility, giving rationale. | 5-7, table 1 |
| Information sources | 7 | Describe all information sources (e.g., databases with dates of coverage, contact with study authors to identify additional studies) in the search and date last searched. | 6 |
| Search | 8 | Present full electronic search strategy for at least one database, including any limits used, such that it could be repeated. | Table 1, Table S2 |
| Study selection | 9 | State the process for selecting studies (i.e., screening, eligibility, included in systematic review, and, if applicable, included in the meta-analysis). | 7 |
| Data collection process | 10 | Describe method of data extraction from reports (e.g., piloted forms, independently, in duplicate) and any processes for obtaining and confirming data from investigators. | N/A |
| Data items | 11 | List and define all variables for which data were sought (e.g., PICOS, funding sources) and any assumptions and simplifications made. | N/A |
| Risk of bias in individual studies | 12 | Describe methods used for assessing risk of bias of individual studies (including specification of whether this was done at the study or outcome level), and how this information is to be used in any data synthesis. | N/A |
| Summary measures | 13 | State the principal summary measures (e.g., risk ratio, difference in means). | N/A |
| Synthesis of results | 14 | Describe the methods of handling data and combining results of studies, if done, including measures of consistency (e.g., I^2^) for each meta-analysis. | N/A |
| Risk of bias across studies | 15 | Specify any assessment of risk of bias that may affect the cumulative evidence (e.g., publication bias, selective reporting within studies). | N/A |
| Additional analyses | 16 | Describe methods of additional analyses (e.g., sensitivity or subgroup analyses, meta-regression), if done, indicating which were pre-specified. | 7-8 |
| **RESULTS** | | |  |
| Study selection | 17 | Give numbers of studies screened, assessed for eligibility, and included in the review, with reasons for exclusions at each stage, ideally with a flow diagram. | 8, Figure 1, Table S3 |
| Study characteristics | 18 | For each study, present characteristics for which data were extracted (e.g., study size, PICOS, follow-up period) and provide the citations. | N/A |
| Risk of bias within studies | 19 | Present data on risk of bias of each study and, if available, any outcome level assessment (see item 12). | N/A |
| Results of individual studies | 20 | For all outcomes considered (benefits or harms), present, for each study: (a) simple summary data for each intervention group (b) effect estimates and confidence intervals, ideally with a forest plot. | N/A |
| Synthesis of results | 21 | Present results of each meta-analysis done, including confidence intervals and measures of consistency. | N/A |
| Risk of bias across studies | 22 | Present results of any assessment of risk of bias across studies (see Item 15). | N/A |
| **Section/topic** | **#** | **Checklist item** | **Reported on page #** |
| Additional analysis | 23 | Give results of additional analyses, if done (e.g., sensitivity or subgroup analyses, meta-regression | 9, table 2, supplementary text |
| **DISCUSSION** | | |  |
| Summary of evidence | 24 | Summarize the main findings including the strength of evidence for each main outcome; consider their relevance to key groups (e.g., healthcare providers, users, and policy makers). | 10 |
| Limitations | 25 | Discuss limitations at study and outcome level (e.g., risk of bias), and at review-level (e.g., incomplete retrieval of identified research, reporting bias). | 10-11 |
| Conclusions | 26 | Provide a general interpretation of the results in the context of other evidence, and implications for future research. | 11-12 |
| **FUNDING** | | |  |
| Funding | 27 | Describe sources of funding for the systematic review and other support (e.g., supply of data); role of funders for the systematic review. | 13 |

*From:*  Moher D, Liberati A, Tetzlaff J, Altman DG, The PRISMA Group (2009). Preferred Reporting Items for Systematic Reviews and Meta-Analyses: The PRISMA Statement. PLoS Med 6(6): e1000097. doi:10.1371/journal.pmed1000097

Table S2: Search terms applied in PubMed

| Area | MeSH thesaurus headings | Free text |
| --- | --- | --- |
| Population | Tuberculosis, Multidrug-Resistant [MeSH] | ((tuberculosis OR TB) AND (multidrug-resistan* OR multidrug resistan* OR multi-drug resistan* OR drug resistan* OR drug-resistan* OR multiresistan* OR “multi resistan*” OR rifampicin resistan* OR isoniazid resistan* OR (drug resistan* NEAR extensive*) OR (drug-resistan* NEAR extensive*) OR MDR OR XDR OR TDR)) OR MDRTB OR XDRTB OR TDRTB OR MDR-TB OR XDR-TB OR TDR-TB OR “MDR TB” OR “XDR TB” OR “TDR TB” |
| Intervention | Time to Treatment [MeSH]  OR  Early Diagnosis [MeSH] | (“treatment start” OR “treatment initiat*” OR "treatment delay" OR "late treatment")) |

Population terms were combined using Boolean operators AND/OR. Intervention terms were combined using OR. Population and intervention term groupings were then be combined using AND. Comparator and outcome terms were not included in the search strategy as the number of hits from combining population and intervention terms was deemed manageable for sifting.

Table S3: Number of search hits by database source

|  | Total items retrieved | After duplicates deleted (n=475)  After pre-1990 excluded (n=28) |
| --- | --- | --- |
| PubMed | 621 | 489 |
| Embase | 382 | 307 |
| Cochrane Library | 133 | 115 |
| WHO Global Index Medicus | 734 | 465 |
| WHO Portal of Clinical Trials | 6 | 6 |
| OpenSIGLE | 2 | 2 |
| Google Scholar | 100 | 91 |
| TOTAL | 1978 | 1475 |

**Supplementary post hoc review text**

### Background to this section

In the absence of any publications fulfilling the protocol inclusion criteria we decided to revisit the 64 articles selected for full text review to determine whether any data could be abstracted that addressed the less constrained question of whether treatment outcome could be related to delay to treatment initiation.

For this we relaxed the requirement for distinct groupings of early or late treatment initiators and considered for inclusion any article that reported time to treatment initiation and that also related treatment outcomes, including interim outcomes such as sputum conversion, to time to treatment initiation. We sought to include articles that might provide data on a gradient of treatment initiation, even if most included patients had suffered a treatment delay.

### General comments

Many studies differentiated between new and previously treated patients; however we did not consider this as a suitable surrogate from which we could infer differential treatment delays. Previous treatment with a larger number of drugs was frequently reported to lead to poorer outcomes, but time to initiation of treatment was usually not considered, and could not be elucidated.

Similarly, several studies divided patients into subgroups with (often loosely defined) advanced and less advanced disease, and whilst the question of whether extent of disease influences treatment outcome is of interest, it is not the question being addressed in this review and cannot be considered as a useful proxy for delay to treatment initiation. A common finding was presentation of time to treatment initiation in a summary statistic for the whole cohort, without disaggregation of this or breakdown of outcome analysis by time to treatment initiation.

### Narrative report of data that may contribute to discussion

There are significant difficulties in coming to any useful conclusions from the available data, as exposures and outcomes are heterogeneous and combining data would not be appropriate, however that data which may at least inform the discourse has been extracted and is presented here in narrative and tabulated format.

Some studies report operational data before and after implementation of specific programmatic management of drug-resistant tuberculosis interventions. Whilst these analyses do not allow for attribution of changes in treatment outcomes to changes in pre-treatment disease duration (treatment outcomes are not reported by treatment delay strata and the before/after design and retrospective nature in any case result in important confounding), nevertheless there are some interesting observations. Mainly these report concurrent reductions in delay to treatment initiation and improvements in various interim treatment outcomes; numerous changes in the programmatic management contribute to improved treatment outcomes such that attribution or independent association with reduction in time to treatment initiation cannot be demonstrated or inferred. Moreover for the majority of papers both treatment initiation delay and treatment outcomes are outcome measures resulting from exposures to differing health systems. Data were abstracted from sixteen articles and they are presented in chronological order in table 2 in the main article.
